# Feasibility of omitting clinical target volume for limited-disease small cell lung cancer treated with chemotherapy and intensity-modulated radiotherapy

**DOI:** 10.1186/1748-717X-9-17

**Published:** 2014-01-10

**Authors:** Shuhua Cai, Anhui Shi, Rong Yu, Guangying Zhu

**Affiliations:** 1 Department of Radiation Oncology, Fujian Provincial Cancer Hospital, Fujian Medical University Teaching Hospital, 420# Fuma Road, Fuzhou 350014, People’s Republic of China; 2 Key Laboratory of Carcinogenesis and Translational Research (Ministry of Education), Department of Radiation Oncology, Peking University Cancer Hospital & Institute, 52# Fucheng Road, Beijing 100142, People’s Republic of China

**Keywords:** Radiotherapy, Lung cancer, Limited-disease small cell lung cancer, Clinical target volume, Local relapse, Intensity-modulated radiotherapy, Radiation pneumonia

## Abstract

**Purpose:**

To analyze the feasibility of omitting clinical target volume (CTV) for limited small cell lung cancer treated with chemotherapy and intensity modulated radiotherapy.

**Methods and materials:**

89 patients were treated from January 1, 2008 to August 31, 2011, 54 cases were irradiated with target volume without CTV, and 35 cases were irradiated with CTV. Both arms were irradiated post chemotherapy tumor extent and omitted elective nodal irradiation; dose prescription was 95% PTV56-63 Gy/28-35 F/5.6-7 weeks.

**Results:**

In the arm without CTV and arm with CTV, the local relapse rates were 16.7% and 17.1% (p = 0.586) respectively. In the arm without CTV, of the 9 patients with local relapse, 6 recurred in-field, 2 recurred in margin, 1 recurred out of field. In the arm with CTV, of the 6 patients with local relapse, 4 recurred in-field, 1 recurred in margin, 1 recurred out of field. The distant metastases rates were 42.6% and 51.4% (p *=* 0.274) respectively. Grade 3-4 hematological toxicity and radiation esophagitis had no statistically significant, but grade 3-4 radiation pneumonia was observed in only 7.4% in the arm without CTV, compared 22.9% in the arm with CTV (p = 0.040). The median survival in the arm without CTV had not reached, compared with 38 months in the with CTV arm. The l- years, 2- years, 3- years survival rates of the arm without CTV and the arm with CTV were 81.0%, 66.2%, 61.5% and 88.6%, 61.7%, 56.6% (p = 0.517). The multivariate analysis indicated that the distant metastases (p = 0.000) and PCI factor (p = 0.004) were significantly related to overall survival.

**Conclusions:**

Target delineation omitting CTV for limited-disease small cell lung cancer received IMRT was feasible. The distant metastases and PCI factor were significantly related to overall survival.

## Background

Lung cancer constitutes the major cause of cancer-related mortality worldwide. Small cell lung cancer (SCLC) accounts for 15%-25% among lung cancer and of which 25% - 40% were limited diseases [1]. About 80-90% of patients with limited disease respond to chemotherapy but relapse generally occurs with median time of 8 months; local recurrence occurs in 90% of patients treated with chemotherapy alone. This led to the use of radiotherapy to local disease in an effort to improve local control and survival. The most common chemotherapy regimen is carboplatin and etoposide. This is also used in concurrent therapy. Radiotherapy should be delivered concurrently with chemotherapy (randomised trial), and with the 1st or 2nd cycle of chemotherapy. The early delivery of concurrent chemotherapy with chest irradiation has become the current treatment standard for LSCLC [2-4]. The lung itself is regarded as a very sensitive organ to radiation damage. In delivering radiation therapy to the lung and mediastinum, attention must be devoted to tolerance of normal tissues. Many publications have addressed the consequences of radiation pneumonia, which might be a life threatening complication [5,6].

Following ICRU 62, the gross tumor volume (GTV) is the volume that contains the visible or clinically detectable tumor, this may be on clinical examination or on imaging. The clinical target volume (CTV) is a tissue volume that contains a GTV and/or subclinical microscopic malignant disease, which has to be eliminated. This volume has to be treated adequately in order to achieve the aim of the therapy: cure or palliation. The internal target volume (ITV) includes a margin to account for physiological patient movements that are unable to be accounted for during treatment. This may include movement of the gut, beating of the heart or respiration. The margin required is known as the internal margin and may vary in height, breadth and depth based on the location within the body. The ITV is a newer concept that attempts to divide treatment inaccuracies into internal patient factors and external factors. If a method to reduce the effect of internal movements is used (eg. respirator gating), then the ITV can be substantially reduced. The plan target volume (PTV) is a geometrical concept, and it is an expansion from the ITV to account for external treatment inaccuracies. These may vary based on the department and the treatment site. This distance is the external margin. Improving the external factors which lead to treatment inaccuracies may reduce the external margin and allow for smaller PTV expansions. Delineation of GTV, CTV, ITV and PTV is now the standard for current intensity-modulated radiotherapy for patients with LSCLC. But RTOG 0617, whose delineation of target volume was accordant with ICRU 62, failed to escalate the dose from 60 Gy to 74 Gy because more toxicities. The radiation oncologists are considering where we should go next. Shrinking the treatment volume with dose escalation may be a reasonable way. Usually the small cell lung cancer is more sensitive to radiation than non-small cell lung cancer. So the study to investigate the feasibility of target delineation omitting CTV was initiated in our department. The following is the preliminary results.

## Materials and methods

### Ethical approval

Feasibility of omitting clinical target volume for limited-disease small cell lung cancer treated with chemotherapy and intensity-modulated radiotherapy.

### Patients

89 patients were treated in the Department of Radiation Oncology at Peking University Cancer Hospital from January 1, 2008 to August 31, 2011. Limited disease being defined as tumor confined to one hemi thorax, including regional lymph nodes, ipsilateral hilar, bilateral mediastinal and bilateral supraclavicular nodes without pleural effusion. The entry criteria were as follows: cytological or histological proven SCLC; WHO performance status 0-1. Staging procedures included as basic laboratory studies, computed tomography (CT) of the chest with contrast, CT or ultrasound imaging of the abdomen, bone emission computerized tomography (ECT) and brain CT or magnetic resonance imaging (MRI). Exclusion criteria were receipt of tumor resection; prior thoracic cancer; thoracic radiation; a diagnosis of other primary cancer (other than skin cancer). All patients in this study were untreated before been recruited.

### Treatment schedule

Conventional simulation CT scans were acquired with 5-mm slices. The pulmonary extent of lung tumors were delineated on pulmonary windows (width 1600 HU, level-600 HU) and the mediastinal lymph nodes were delineated on mediastinal windows (width 400 HU, level 20 HU).

In the with CTV arm, target volume was delineated according to the definition of ICRU62. The GTV was delineated according to CT or PET, a lymph node was considered to be involved with tumor if it was positive on biopsy or on PET or was ≥10 mm in the short axis on CT. A margin of 8 mm to cover microscopic spread of disease was added to GTV to form the CTV. A 3- to 15-mm margin was added to create an ITV to cover respiratory movement; respiratory movement for every patient was measured on conventional simulator, not 4D CT during free breathing. A 5-mm margin was added to create the PTV considering setup variations.

In the without CTV arm, the CTV was omitted, the GTV, ITV and PTV was delineated at the same way with the control arm.

Both arms were irradiated post chemotherapy tumor extent if received Induction chemotherapy and omitted elective nodal irradiation. Dose prescription was 95% PTV56-63 Gy/28-35 F/5.6-7 weeks.

The IMRT plans were developed by using a commercial treatment-planning system (Varian Medical Systems). Normal structures delineated included lung (excluding the GTV from the rest of the lung parenchyma), heart, esophagus, and spinal cord with a 5-mm margin as the planning organ-at-risk margin. Patients who achieved partly remission (PR) of tumor after the completion of chemoradiotherapy were offered prophylactic cranial irradiation (PCI), however, some patients failed to followed doctor’s advices or refused to receive cranial irradiation , only 46 patients underwent PCI. Dose prescription was PTV 25 Gy/10F/2 weeks.

### Follow-up

Patients were followed up at regular intervals, usually every 3-4 months for the first 2 years after treatment, then every 6 months during 3-5 years. Follow-up examinations included basic laboratory studies, liver and renal function tests, CT of the chest, CT or MRI of the brain, and positron emission tomography (PET) when needed. Relapse in-margin was defined as the region 5 mm inside and outside of PTV. Relapse in-field and out-of-field were defined as in and out the area of relapse in-margin respectively. The final follow-up time was June 30, 2012.

### Statistical analysis

Statistical analysis was performed with SPSS 17.0. Overall survival, progression free survival and local relapse free survival were calculated for all patients on an intention-to-treat basis, using the Kaplan–Meier method and starting from the first day of the treatment. Patients lost to follow-up or alive at the time of analysis were censored at the time of last follow-up.

The differences between the two arms were assessed using λ^2^-test or Fisher’s exact tests for the categorical outcome variables. The influence of variables for survival was studied by Kaplan-Meier analysis and Cox regression analysis.

## Results

From January 1, 2008 to August 31, 2011, in the Department of Radiation Oncology at Peking University Cancer Hospital, 89 patients were evaluated in the study, 54 cases were irradiated with target volume without CTV, 35 cases with target volume with CTV. Patients characteristics were listed in Table 1.

**Table 1:** Patients characteristics

**Characteristic**	**Arm without CTV n (%)**	**Arm with CTV n (%)**	***p*-value**
No. of eligible patients	54(100)	35(100)	
Sex			0.339
Male	41(75.9)	23(42.6)	
Female	13(24.1)	12(57.4)	
Age (y)			
Median	56	57	0.548
Range	36-78	38-76	
≤65 years	47(87.0)	30(85.7)	
>65 years	7(13.0)	5(14.3)	
Performance score			0.350
0	32(59.3)	23(65.7)	
1	22(40.7)	12(34.3)	
Weight loss			0.600
≤5%	46(85.2)	30(85.7)	
>5%	8(14.8)	5(14.3)	

The cisplatin plus etoposide regimen was the standard scheme for SCLC [7]. In the study, the arm with CTV accepted 1.87 ± 1.56 cycle induction chemotherapy, 1.65 ± 0.81 cycle concurrent chemotherapy, 0.59 ± 0.92 cycle adjuvant chemotherapy; the arm without CTV accepted 2.4 ± 1.63 cycle induction chemotherapy, 1.63 ± 1.09 cycle synchronous chemotherapy, 0.83 ± 1.07 cycle adjuvant chemotherapy. Treatment delivery was listed in Table 2.

**Table 2:** Treatment delivery

**Characteristic**	**Arm without CTV n (%)**	**Arm with CTV n (%)**
No. of eligible patients	54(100)	35(100)
Treatment schedule		
Induction chemo + RT	6(11.1)	8(22.9)
Concurrent chemo alone	2(3.7)	2(5.7)
Induction + concurrent t adjuvant chemo	11(20.4)	13(37.1)
Induction + concurrent chemo	26(48.1)	10(28.6)
Concurrent + adjuvant chemo	8(14.8)	2(5.7)
Induction + RT adjuvant chemo	1(1.9)	0(0)
Induction chemo (cycles)		
0	10(18.5)	4(11.4)
1	15(27.8)	9(25.7)
2	13(24.1)	7(20.0)
≥3	16(29.6)	15(42.9)
Concurrent chemo (cycles)		
0	7(13.0)	8(22.8)
1	9(16.7)	5(14.3)
2	34(63.0)	14(40.0)
3	4(7.4)	8(22.8)
Adjuvant chemo (cycles)		
0	34(63.0)	20(57.1)
1	8(14.8)	5(14.3)
2	11(20.4)	9(25.7)
3	1(1.9)	1(2.9)
PCI		
Without PCI	25(46.3)	18(51.4)
With PCI	29(53.7)	17(48.6)

### Response and survival

The volumes and dosage of GTV and PTV and short-term response rate of both arms were listed in Table 3.

**Table 3:** The volumes and dosage of GTV and PTV and short-term response rate of both arms

**Characteristic**	**Arm without CTV**	**Arm with CTV**	***p*-value**
Volumes (cm^3^)			
GTV	38.3 ± 5.0	37.3 ± 3.8	0.329
PTV	199.2 ± 19.1	327.4 ± 26.5	0.000
Dosage(Gy)			
GTV	67.1 ± 2.0	66.7 ± 2.5	0.386
PTV	61.2 ± 2.0	60.8 ± 2.2	0.328
Short-term response			0.973
CR	18	12	
PR	29	18	
SD	7	5	
PD	0	0	

Analysis of survival data was made after median follow-up of 20 months (range 6-53 months). In the whole 89 cases, local relapse occurred in 15 out of 89 patients (16.6%), distant metastasis 41 out of 89 patients (46.1%), l years, 2 years, 3 years survival rates were 79%, 63%, 53%, the median survival time was 51.3 months.

In the arm without CTV and with CTV, the local relapse rates were 16.7% and 17.1% (p = 0.586), the distant metastases rates were 42.6% and 51.4% (p = 0.274) respectively, difference between local relapses and distant metastasis had no statistically significant. The sites of relapse were listed in Table 4.

**Table 4:** Site of relapse of LSCLC patients receiving chemotherapy and IMRT

**Characteristic**	**Arm without CTV n (%)**	**Arm with CTV n (%)**
No. of eligible patients	54(100)	35(100)
Relapse site		
Local- relapse #	9(16.7)	6(17.1)
In-field	6(11.1)	4(11.3)
In-margin	2(3.7)	1(2.9)
Out-of-field	1(1.9)	1(2.9)
Distant metastasis*	23(42.6)	18(51.4)
Distant alone	17(31.5)	13(37.1)
Local + distant	6(11.1)	5(14.3)
Metastasis sites		
Brain	11(20.4)	7(20.0)
Bone	8(14.8)	6(17.1)
Liver	8(14.8)	3(8.6)
Lung	4(7.4)	2(5.7)
Adrenal gland	3(5.6)	1(2.9)
Peritoneum LN	2(3.7)	3(8.6)

# p = 0.586 *p = 0.274.

**Figure 1:**
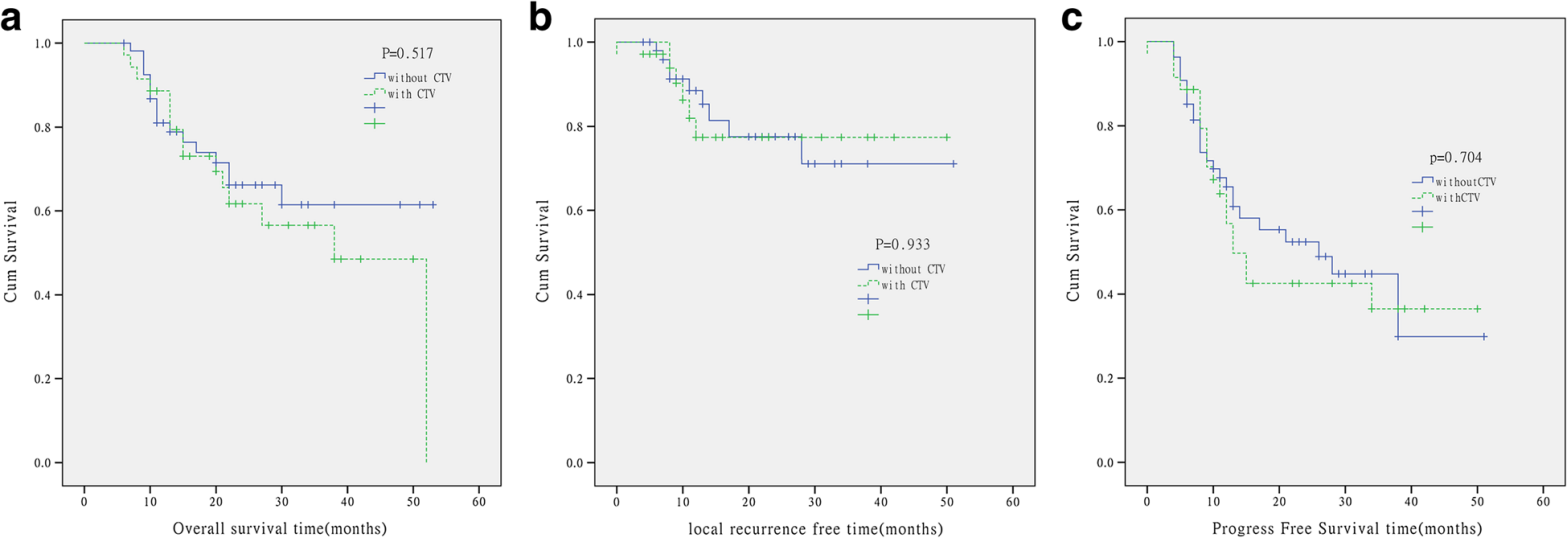
Kaplan-Meier analysis of overall survival (a), local relapse free survival (b) and progress free survival (c) for the patients target delineation without CTV and with CTV in limited small cell lung cancer.

The median overall survival time in the without CTV arm hadn’t reached, compared with 38.0 months (95%CI: 28.4-47.6) in the with CTV arm. The l- years, 2- years, 3- years overall survival rates of without CTV arm and with CTV arm were 81.0%, 66.2%, 61.5% and 88.6%, 61.7%, 56.6% (p = 0.517),the overall survival had no statistically significant difference between both arms (Figure 1a). The difference in local relapse free survival and distant metastasis free survival between without CTV arm and with CTV arm had no statistically significant (p = 0.933 and p = 0.565) (Figure 1b and c).

**Figure 2:**
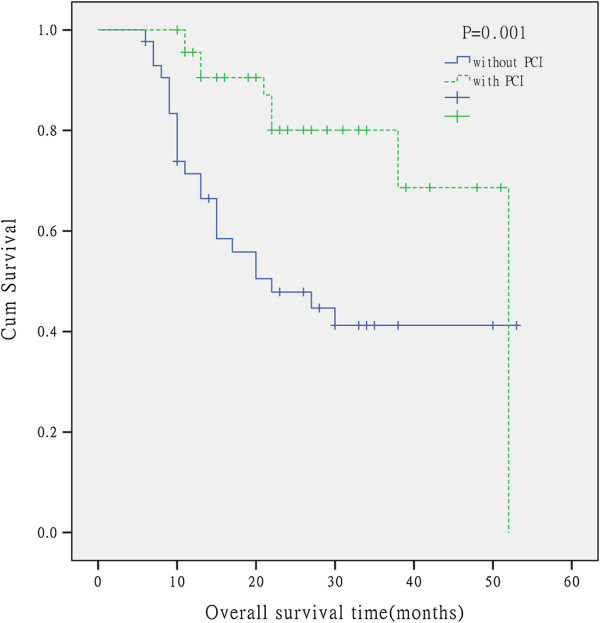
Kaplan-Meier analysis of overall survival for the patients without PCI and with PCI in limited small cell lung cancer.

46 patients were administered prophylactic cranial irradiation (PCI), dose prescription was PTV25 Gy/10F/2 weeks, of which 3 cases had accident of brain metastasis. Other 43 patients hadn’t received PCI, 15 case had accident of brain metastasis (p = 0.001). The median survival in with PCI arm was 52 months, compared with 22 months in the without PCI arm. LSCLC treated with PCI decreased brain metastases and benefited the survival time. The difference between with PCI and without PCI had statistically significant (p = 0.001) (Figure 2).

Univariate analysis and subsequently multivariate analyses were performed. No pre-treatment factor was significantly related to overall survival. The multivariate analysis indicated that the distant metastases (p = 0.000) and PCI factor (p = 0.004) were significantly related to overall survival by Cox regression.

### Radiation-related toxicity

Radiation-related toxicity was scored according to the Common Terminology Criteria for Adverse Events (CTCAE 3.0). In neither arm was a treatment-related death observed. The treatment-related toxicity was summarized in Table 5.

**Table 5:** Toxicities experienced of LSCLC patients receiving chemotherapy and IMRT

**Toxicities**	**Arm without CTV n(%)**	**Arm with CTV n(%)**	***p-value***
Hematologic toxicity			0.485
0-2	40 (74.1)	25(71.4)	
3-4	14 (25.9)	10(28.6)	
Radiation esophagitis			0.123
0-2	40(74.1)	21(60.0)	
3-4	14(25.9)	14(40.0)	
Radiation pneumonia			0.040
0-2	51(92.6)	27(77.1)	
3-4	4(7.4)	8(22.9)	

## Discussion

IMRT has improved the ability to deliver higher radiation doses to tumors while spare surrounding normal structures. Many advances have been achieved nowadays. These include CT-based treatment planning, conformal radiation therapy, positron emission tomography and knowledge of tumor motion during radiation delivery. So it has become more common in the treatment of SCLC. A trend in modern thoracic radiotherapy is toward more conformal fields. Emerging clinical data show that omitting prophylactic lymph node irradiation for Limited-Stage SCLC did not reduce the local control rate for patients receiving thoracic radiotherapy, the isolated outside-field local recurrence rates were less than 8% [8,9]. The rate of isolated nodal failure not using elective nodal irradiation (ENI) was similar to what reported in studies using ENI [10-12]. Hu X [13] preliminary results indicated that irradiated post chemotherapy tumor extent and omitted elective nodal irradiation did not decrease local regional control and the overall survival difference was not statistically significant. The local recurrence rates were 31.6% (12 of 38) and 28.6% (12 of 42), respectively (p =0.81). The isolated nodal failure rates were 2.6% (1 of 38) and 2.4% (1 of 42), respectively (p =1.00). All the effort was to reduce the irradiation volumes then reduce the radiation injury and do not decrease local regional control at the same time.

The RTOG 0617 trial involved 419 patients with stage III NSCLC, and compared high-dose (74 Gy) with standard-dose (60 Gy) radiation. All patients also received chemotherapy with paclitaxel and carboplatin. From the interim results, we saw that the higher dose of radiation (74 Gy) was associated with a higher rate of serious (grade 3) esophagitis (21% vs. 7%, p = 0.0003) and more treatment-related deaths (10 vs. 2).Compared high-dose with standard-dose , the PFS was 26.3% vs. 36.6% (p = 0.0116) and the OS was 53.9% vs. 66.9% at 18 months, median OS 19.5 vs. 28.7 months (p = 0.0007). The results Were Surprising. It is possible that the high dose increased radiation to the heart, extended therapy, or caused unreported toxicity, or it could be a combination of these factors. So we cannot elevate doses in spite of radiation toxicity.

What is the next step? Can we treat LSCLC with smaller tumor volume using IMRT? Is CTV necessary? CTV includes the GTV plus a margin to encompass subclinical or microscopic malignant disease immediately adjacent to GTV. In lung cancer, a study [14] demonstrated that GTV-to-CTV expansions of 6 mm for squamous carcinoma and 8 mm for adenocarcinoma are required to cover the gross tumor and microscopic disease. Expansions for SCLC have not been determined, but a conservative approach would be to use 8 mm because of the character of its invasive growth.

Obviously, thoracic radiotherapy omitting CTV reduced the radiation volume, what we concerned most was whether omitting CTV result in high local relapse rate. In the study, the local relapse rates were 16.7% in arm without CTV and 17.1% in arm with CTV. Van Loon et al evaluated the impact of PET scan usage on selective nodal irradiation in patients with LSCLC and their results showed a low isolated nodal failure rate of 3% [15,16]. The major local relapse was in-field local relapse, the in-field recurrences were presented in the place where hadn’t reached complete response in the initial region. The possible reason was the existing of radiation-resisted cancer cell which need higher irradiation doses to enhance local control [17,18]. Currently ongoing trial compares 45 Gy twice-daily dose with a 2 Gy once-daily to 66-70 Gy concomitantly with EP regimen chemotherapy. We look forward to seeing whether the higher dose result in the improvement of local control and long-term survival.

In the study, with the reduction of the thoracic radiation volume in the arm without CTV, grade 3-4 radiation esophagitis occurred in 25.9% while 40.0% in the arm with CTV (p = 0.123). Grade 3-4 acute radiation pneumonia was more pronounced in 22.9% of patients in the arm with CTV, while it was observed in only 7.4% in the arm without CTV (p = 0.040). Thoracic radiotherapy omitting CTV had decreased esophageal and lung toxicity, improve the patients quality of life, which suggests the possibility of dose escalation and allows for concurrent systemic chemoradiotherapy in a greater proportion of patients.

To the best of our knowledge, this was the first clinical study reporting target delineation without CTV in LSCLC. There were several possible reasons for this philosophy. First, SCLC is an aggressive type of lung cancer characterized by rapid growth and early distant metastasis; in the study, 16.6% patients suffer local relapse, 46.1% patients suffer distant metastasis, study indicated that the distant metastases and PCI factor were significantly related to overall survival. Patients would have died of distant metastasis before the local failure became clinically apparent. It was the distant metastasis which eventually leads to death; by contrast, local relapse was the subordinate factor in the cause of death. If distant metastases cannot be controlled, what is the meaning of perfect local disease control? Enlarged radiation field only lead to more damage produced by radiation. Secondly, micro-metastasis in CTV may have been small enough to be eliminated by effective chemotherapy due to the sensitivity of SCLC to chemotherapy. Furthermore the tumor cells scattered in CTV were in oxygen enrichment condition and had better radiation sensitivity [19].In the arm without CTV, the dose of PTV reach 62 Gy, at the region of 8 mm expansion of the PTV,the mean dose reach 32 Gy in dosimetric report. Study [20,21] indicated that incidental dose to the ipsilateral hilum, paratracheal and mediastinal nodes approach 40-50 Gy when these regions were not intentionally irradiated. The incidental dose was enough to eliminate the microscopic spread cancer cells.

## Conclusion

In conclusion, the preliminary results indicated that thoracic radiotherapy omitting CTV for limited-disease small cell lung cancer did not increase loco-regional failure while significantly fewer patients suffered from grade 3-4 acute radiation pneumonia. So we concluded that target delineation omitting CTV for limited-disease small cell lung cancer received IMRT may be feasible. Further investigation is warranted.

## Abbreviations

GTV: Gross tumor volume; CTV: Clinical target volume; ITV: Internal target volume; PTV: Plan target volume; SCLC: Small cell lung cancer; LSCLC: Limited-disease small cell lung cancer; CT: Computed tomography; ECT: Emission computerized tomography; MRI: Magnetic resonance imaging; PET: Positron emission tomography; PCI: Prophylactic cranial irradiation; ENI: Elective nodal irradiation; CR: Complete remission; PR: Partial response; SD: Stable disease; PD: Progressive disease.

## Competing interests

The authors have declared that no competing interests exist.

## Authors’ contributions

SC selected the data and performed the analysis, drafted and wrote the manuscript. AS and RY were responsible for patient treatment and care. GZ contributed substantially to conception and design, reviewed and revised the manuscript. All authors read and approved the final manuscript.
